# Agreeing to disagree: reports of the popularity of Covid-19 conspiracy theories are greatly exaggerated

**DOI:** 10.1017/S0033291720002780

**Published:** 2020-07-20

**Authors:** Robbie M. Sutton, Karen M. Douglas

**Affiliations:** University of Kent, UK

A study recently published in this journal showed that agreement with conspiracy theories about the Covid-19 pandemic is associated with risky, non-compliant behaviours (Freeman et al., 2020a). It also indicated that this agreement is very common: 45% of British participants seemed to agree that Covid-19 is a bioweapon designed by China to destroy the West, while 20% seemed to agree that the pandemic is a conspiracy by Jews or Muslims. Accurate or not, these statistics paint a worrying picture. If accurate, millions of British people need to be disabused of wild conspiracy theories. If inaccurate, especially if they exaggerate the popularity of conspiracy theories, they could normalise Antisemitic, Islamaphobic, and conspiracist viewpoints (McManus, D'Ardenne, & Wessely, 2020), and misdirect policy, interventions, and further research.

McManus et al. (2020) pointed out that Freeman et al.'s (2020a) study indeed runs these risks, because of a response scale that gave participants four options to agree (from ‘agree a little’ to ‘agree completely’), and only one other option (‘do not agree’). This imbalance of options is likely to cause participants who tend to acquiesce to perceived demands of survey questions to report inflated levels of agreement (Hibbing, Cawvey, Deol, Bloeser, & Mondak, 2019).

We agree with this critique. As researchers who have published many papers on conspiracy theories, including their conceptualisation and measurement (Douglas, Sutton, & Cichocka, 2017; Douglas et al., 2019; Douglas & Sutton, 2018; Lantian, Muller, Nurra, & Douglas, 2016; Sutton & Douglas, 2020), we do not recall seeing a scale like Freeman et al.'s (2020a). Scales typically provide an even balance of responses to reject or accept conspiracy theories. This allows participants to express any view on the assumed continuum between strong disagreement and strong agreement. Since responses are typically below or near the midpoint on such scales (e.g. Imhoff and Lamberty, 2017; Jolley and Douglas, 2014), omitting degrees of disagreement seems an important mistake. Participants who disagree with a conspiracy theory, but are willing to admit that it might have some merit, may feel that they have no option but to select one of the ‘agree’ responses. This hypothetical dilemma lends new meaning to the saying ‘agreeing to disagree’.

To test the hypothesis that Freeman et al.'s (2020a) scale exaggerates agreement with conspiracy theories, we ran a brief, pre-registered study. Materials, anonymised data, and results are available on the Open Science Framework website: https://osf.io/xpvrz. We chose three conspiracy theories from Freeman et al., targeting Jews, Muslims, and China, that featured prominently in a press release (University of Oxford, 2020) and attracted media attention. We presented each to 748 British participants recruited from Prolific, a widely used survey platform (Peer, Brandimarte, Samat, & Acquisiti, 2017), who were British nationals resident in the UK aged 18 or over, and not currently students since this group is over-represented on Prolific. Their age ranged from 18 to 80 (*M* = 38.75, s.d. = 12.70); 506 were female, 238 male, and 4 were gender queer; 681 were White, 18 Black, 29 Asian, and 20 were mixed race.

Participants were then randomly assigned to three groups. The first group (*n* = 251) were given Freeman et al.'s (2020a) response scale. The second (*n* = 251) responded on a conventional five-point scale featuring two options to disagree, two to agree, and a ‘neither agree nor disagree’ option (see Douglas et al., 2019 for a summary of different conspiracy belief measures). The third group (*n* = 246) responded on a nine-point scale constructed by mirroring each of the four agreement responses in Freeman et al.'s (2020a) study with a corresponding disagreement response, and included a ‘neither agree nor disagree’ option.

Following Freeman et al. (2020a, b) and our pre-registration, we coded as agreement (1) the four response options expressing agreement on Freeman et al.'s scale and the nine-point extension, and either of the options expressing agreement on the conventional five-point scale. Responses were otherwise coded as not expressing agreement (0). Thus, across the three conspiracy theories, participants could score between 0 (agreed with none) and 3 (agreed with all).

All conspiracy theories, response options and response proportions are presented in Table 1. It reveals strikingly lower rates of agreement than in Freeman et al. (2020a). Even on the same response scale, 2% or 3% of our participants agreed with the conspiracy theories about Jews and Muslims (compared to 20% in Freeman et al.), and 32% (compared to 45%) agreed with the China theory. These differences between studies were expected (see pre-registration) and significant (*p*s < 0.001). Their magnitude is surprising and noteworthy, but also difficult to interpret since the studies differ in many ways. For example, our study was run in late June 2020 and Freeman et al.'s study was run in early May; ours used a relatively educated sample, among whom slightly lower agreement with conspiracy theories can be expected (Douglas, Sutton, Callan, Dawtry, & Harvey, 2016).

**Table 1.** Proportion of respondents selecting each response to the three conspiracy theories

	Freeman et al. (2020a) response scale (*N* = 251)					
	Do not agree	Agree a little	Agree moderately	Agree a lot	Agree completely	*Sum agreement*
Jews have created the virus to collapse the economy for financial gain	98.0%	1.2%	0.8%	0.0%	0%	2.0%
Muslims are spreading the virus as an attack on Western values	97.2%	1.6%	0.4%	0.8%	0%	2.8%
Coronavirus is a bioweapon developed by China to destroy the West	68.1%	21.1%	8.8%	1.6%	0.4%	31.9%

	Five-point balanced response scale (*N* = 251)					
	Strongly disagree	Disagree	Neither agree nor disagree	Agree	Strongly agree	*Sum agreement*
Jews have created the virus to collapse the economy for financial gain	90.8%	6.8%	2.0%	0%	0.4%	0.4%
Muslims are spreading the virus as an attack on Western values	86.9%	10.4%	1.2%	0.4%	1.2%	1.6%
Coronavirus is a bioweapon developed by China to destroy the West	55.8%	22.7%	12.7%	8.0%	0.8%	8.8%

	Nine-point balanced response scale (*N* = 246)					
	No agreement	Agree a little	Agree moderately	Agree a lot	Agree completely	*Sum agreement*
Jews have created the virus to collapse the economy for financial gain	99.2%	0%	0.4%	0%	0.4%	0.8%
Muslims are spreading the virus as an attack on Western values	99.2%	0.4%	0%	0%	0.4%	0.8%
Coronavirus is a bioweapon developed by China to destroy the West	89.4%	6.5%	2.8%	0.4%	0.8%	10.6%

*Note:* Responses coded as agreement are shaded. ‘Sum agreement’ represents the sum of these responses. For comparison, Freeman et al. (2020a) report 19.2% sum agreement for the Jewish conspiracy theory, 19.9% with the Muslim conspiracy theory, and 45.4% with the China conspiracy theory. For the nine-point response scale, ‘No agreement’ subsumes the first five responses (*Disagree completely*, *Disagree a lot*, *Disagree moderately*, *Disagree a little*, *Neither agree nor disagree*). For the Jewish, Muslim, and China conspiracy theories, the ‘Neither agree nor disagree’ option on the nine-point scale was selected by 1.6%, 2.4%, and 8.9% of participants, respectively. The remaining responses were disagree responses.

More pertinent, we found levels of agreement half as low again, or lower, when we used conventional agree−disagree scales. Agreement with the China conspiracy theory reduced to roughly 10%; agreement with the conspiracy theories about Jews and Muslims fell to around 1–1.5%. The levels of agreement on the five-point and nine-point agree−disagree scales were not significantly different from each other, *p* = 0.712, but were significantly lower than on Freeman et al.'s scale (both *p*s < 0.001).

Our results suggest that Freeman et al.'s (2020a) estimates of the popularity of Covid-19 conspiracy theories were overestimated. In their reply to McManus et al. (2020), Freeman et al. (2020b) wrote that, ‘the item content, not the scale, seems to us to merit the real focus’, but in our study the scale doubled the apparent popularity of the item content. As happens often (Lee, Sutton, & Hartley, 2016), striking results of Freeman et al.'s (2020a) study were highlighted in a press release that stripped them of nuance and caveats, and led to some sensational and misleading media reporting that may have complicated the very problems that we all, as researchers, are trying to help solve.

## References

[ref1] Douglas, K. M., & Sutton, R. M. (2018). Why conspiracy theories matter: A social psychological analysis. European Review of Social Psychology, 29(1), 256–298. doi:10.1080/10463283.2018.1537428.

[ref2] Douglas, K. M., Sutton, R. M., Callan, M. J., Dawtry, R. J., & Harvey, A. J. (2016). Someone is pulling the strings: Hypersensitive agency detection and belief in conspiracy theories. Thinking and Reasoning, 22(1), 57–77. doi:10.1080/13546783.2015.1051586.

[ref3] Douglas, K., Sutton, R., & Cichocka, A. (2017). The psychology of conspiracy theories. Current Directions in Psychological Science, 26(6), 538–542. doi:10.1177/0963721417718261. 29276345 PMC5724570

[ref4] Douglas, K. M., Uscinski, J., Sutton, R. M., Cichocka, A., Nefes, T., Ang, J., & Deravi, F. (2019). Understanding conspiracy theories. Advances in Political Psychology, 40(S1), 3–35. doi:10.1111/pops.12568.

[ref5] Freeman, D., Waite, F., Rosebrock, L., Petit, A., Causier, C., East, A., … Lambe, S. (2020a). Coronavirus conspiracy beliefs, mistrust, and compliance with government guidelines in England. Preprint. Psychological Medicine, 21, 1–13. doi:10.1017/S0033291720001890. PMC7264452 32436485

[ref6] Freeman, D., Waite, F., Rosebrock, L., Petit, A., Causier, C., East, A., … Lambe, S. (2020b). We should beware of ignoring uncomfortable possible truths (a reply to McManus *et al*.). Preprint. Psychological Medicine, 21, 1 10.1017/S0033291720002196. PMC7298150 32507116

[ref7] Hibbing, H. V., Cawvey, M., Deol, R., Bloeser, A. J., & Mondak, J. J. (2019). The relationship between personality and response patterns on public opinion surveys: The big five, extreme response style, and acquiescence response style. International Journal of Public Opinion Research, 31(1), 161–177. doi:10.1093/ijpor/edx005.

[ref8] Imhoff, R., & Lamberty, P. K. (2017). Too special to be duped: Need for uniqueness motivated conspiracy beliefs. European Journal of Social Psychology, 47(6), 724–734. doi:10.1002/ejsp.2265.

[ref9] Jolley, D., & Douglas, K. M. (2014). The effects of anti-vaccine conspiracy theories on vaccination intentions. PLoS One, 9(2), e89177. doi:10.1371/journal.pone.0089177. 24586574 PMC3930676

[ref10] Lantian, A., Muller, D., Nurra, C., & Douglas, K. M. (2016). Measuring belief in conspiracy theories: Validation of a French and English single-item scale. International Review of Social Psychology, 29(1), 1–14. doi:10.5334/irsp.8.

[ref11] Lee, E., Sutton, R. M., & Hartley, B. L. (2016). From scientific article to press release to media coverage: Advocating alcohol abstinence and democratising risk in a story about alcohol and pregnancy. Health, Risk & Society, 18, 247–269. doi:10.1080/13698575.2016.1229758. PMC5351785 28367068

[ref12] McManus, S., D'Ardenne, J., & Wessely, S. (2020). Covid conspiracies: Misleading evidence can be more damaging than no evidence at all. Preprint. Psychological Medicine, 21, 1–2. doi:10.1017/S0033291720002184. PMC7298093 32498747

[ref13] Peer, Y., Brandimarte, L., Samat, S., & Acquisiti, A. (2017). Beyond the Turk: Alternative platforms for crowdsourcing behavioral research. Journal of Experimental Social Psychology, 70, 13–163. doi:10.1016/j.jesp.2017.01.006.

[ref14] Sutton, R. M., & Douglas, K. M. (2020). Conspiracy theories and the conspiracy mindset: Implications for political ideology. Current Opinion in Behavioral Sciences, 34, 118–122. doi:10.1016/j.cobeha.2020.02.015.

[ref15] University of Oxford (2020, May 29). Conspiracy beliefs reduce the following of government coronavirus guidance. Retrieved from: http://www.ox.ac.uk/news/2020-05-22-conspiracy-beliefs-reduces-following-government-coronavirus-guidance.

